# Rejuvenating classical brain electrophysiology source localization methods with spatial graph Fourier filters for source extents estimation

**DOI:** 10.1186/s40708-024-00221-2

**Published:** 2024-03-12

**Authors:** Shihao Yang, Meng Jiao, Jing Xiang, Neel Fotedar, Hai Sun, Feng Liu

**Affiliations:** 1 School of Systems and Enterprises, Stevens Institute of Technology, Hoboken, NJ 07030 USA; 2 Semcer Center for Healthcare Innovation, Stevens Institute of Technology, Hoboken, NJ 07030 USA; 3 MEG Center, Division of Neurology, Cincinnati Children’s Hospital Medical Center, Cincinnati, OH 45229 USA; 4 Epilepsy Center, Neurological Institute, University Hospitals Cleveland Medical Center, Cleveland, OH 44106 USA; 5 Department of Neurology, Case Western Reserve University School of Medicine, Cleveland, OH 44106 USA; 6 Department of Neurosurgery, Rutgers Robert Wood Johnson Medical School of Rutgers University, Brunswick, NJ 08901 USA

**Keywords:** EEG/MEG source imaging, Inverse problem, Graph signal processing, Spatial graph filter

## Abstract

EEG/MEG source imaging (ESI) aims to find the underlying brain sources to explain the observed EEG or MEG measurement. Multiple classical approaches have been proposed to solve the ESI problem based on different neurophysiological assumptions. To support clinical decision-making, it is important to estimate not only the exact location of the source signal but also the extended source activation regions. Existing methods may render over-diffuse or sparse solutions, which limit the source extent estimation accuracy. In this work, we leverage the graph structures defined in the 3D mesh of the brain and the spatial graph Fourier transform (GFT) to decompose the spatial graph structure into sub-spaces of low-, medium-, and high-frequency basis. We propose to use the low-frequency basis of spatial graph filters to approximate the extended areas of brain activation and embed the GFT into the classical ESI methods. We validated the classical source localization methods with the corresponding improved version using GFT in both synthetic data and real data. We found the proposed method can effectively reconstruct focal source patterns and significantly improve the performance compared to the classical algorithms.

## Introduction

EEG/MEG are non-invasive measurement modalities with high temporal resolution up to 1 ms. EEG/MEG signals can be collected noninvasively through electrodes or sensors on the scalp. Importantly, EEG/MEG are the direct measurement methods to detect the instantaneous electrophysiological activities of the brain [1]. EEG and MEG are recognized as powerful tools for capturing real-time brain functions by measuring neuronal processes with broad clinical and neuroscience applications [2]. The EEG/MEG source localization aims to solve an inverse problem to reconstruct the underlying electrophysiological brain activities given the measured EEG/MEG signal and the brain forward model [3]. The EEG/MEG source localization is also referred to as Electrophysiological Source Imaging (ESI) [4]. However, the number of EEG/MEG channels is far less than that of the brain sources, which makes the ESI an ill-posed problem.

In the past decades, numerous algorithms have been developed with different assumptions on the configuration of the source signal. One seminal work is minimum norm estimate (MNE) where $\ell _2$ norm is used as a regularization [5], which is to explain the observed signal using a potential solution with the minimum energy. Different variants of the MNE algorithm include dynamic statistical parametric mapping (dSPM) [6] and standardized low-resolution brain electromagnetic tomography (sLORETA) [7]. The $\ell _2$-norm-based methods tend to render spatially diffuse source estimation. To promote sparse solutions, Uutela et al. [8] introduced the $\ell _1$-norm, known as minimum current estimate (MCE). Rao and Kreutz-Delgado proposed an affine scaling method [9] for a sparse ESI solution. The focal underdetermined system solution (FOCUSS) proposed by Gorodnitsky et al. encourages a sparse solution by introducing the $\ell _{p}$-norm regularization [10]. Besides, Bore et al. proposed to use the $\ell _{p}$-norm regularization ($p < 1$) on the source signal and the $\ell _1$ norm on the data fitting error term to achieve sparsity [11]. Babadi et al. demonstrated that sparsely distributed solutions to event-related stimuli could be found using a greedy subspace-pursuit algorithm [12]. Wipf et al. proposed a unified Bayesian learning method that can automatically calculate the hyperparameters for the inverse problems under an empirical Bayesian framework, and the sparsity of the solution is also guaranteed [13]. To mitigate the high level of noise when estimating source signal, Hashemi et al. applied a hierarchical Bayesian (type-II maximum likelihood) model for joint estimation of latent variables for both source and noise [14]. An innovative robust empirical Bayesian framework proposed by Ghosh et al. can estimate the low-rank noise covariance and sparse brain source activity at the same time [15]. Wan et al. reformulate the ESI problem into a graph search problem by exploiting the graph neighborhood information in the brain source space and the optimal solution can be theoretically guaranteed under high noise circumstances [16].

In addition to using optimization approaches, deep learning methods have become increasingly popular in recent years. Hecker et al. came up with a CNN model with a special design of the input matrices of EEG and MEG signal, termed as ConvDip, for source localization which achieved good performance [17]. Jiao et al. proposed to use of a graph Fourier transform based bidirectional long-short-term memory, termed as GFT-BiLSTM, which can reduce the output dimensions and can construct an extended brain region activation. The GFT-BiLSTM method achieves good performance localizing seizure onset zone [18]. To take advantage of both the high spatial resolution of fMRI and the high temporal resolution of EEG, Liu et al. proposed a hierarchical deep transcoding model for fusing simultaneous EEG-fMRI data to estimate the brain source activity with high spatiotemporal resolution [19, 20]. Another multi-modal deep learning model that fuses both MEG and EEG information is shown to have high accuracy compared to using a single modality of EEG or MEG in the context of deep learning approach [21].

As the brain sources are not activated discretely due to its volume conductivity property, a compact and extended area of source estimation is preferred [4, 22, 23], and it has been used for multiple applications, such as somatosensory cortical mapping [24], and epileptogenic zone in focal epilepsy patients [25, 26]. To estimate an extended area of source activation, we propose to use the GFT technique, which has been shown with an improved accuracy in reconstructing extended source activations [18, 27]. GFT plays a pivotal role in understanding the spectral characteristics of the signals on a graph, analogous to how the Fourier Transform reveals the frequency content of a signal in the time domain [28]. By decomposing graph data into its spectral components, it provides a unique perspective on the underlying structure and connectivity/continuity patterns within the graph. Numerous interesting applications can be found in various domains. For example, in their seminal work, Defferrard et al. proposed to use GFT with deep learning and came up with ChebNet, which highlighted the use of GFT in neural networks for graph-structured data [29]. GFT has been used to characterize the spectrum properties of the brain connectivity networks [30], Brahim and Farrugia showed that using GFT-based structural connectivity and functional connectivity analysis can provide a more accurate classification for patients with Autism Spectrum Disorders [31].

In this work, we propose to improve the classical ESI methods using GFT and its low-frequency components as source space subspaces, and we highlight the importance of using GFT for an extended area of source activation before applying the classical source imaging methods. A preliminary version has been published in the Brain Informatics Proceedings [32].

## Method

In this section, we start with the introduction of the ESI inverse problem, which is followed by the presentation of the GFT and the improved version of classical methods using GFT.

### EEG/MEG source imaging

The source imaging forward problem can be described in the formula as $Y = KS + E$, where $Y\in \mathbb {R}^{C\times T}$ is the EEG or MEG measurements, *C* is the number of EEG or MEG channels, *T* represents the time sequence length, $K\in \mathbb {R}^{C\times N}$ is the *leadfield* matrix which is a linear mapping from the brain sources to the EEG/MEG sensors, *N* is the number of brain source, $S\in \mathbb {R}^{N\times T}$ represents the brain source signal in *N* source locations for all the *T* time points, and *E* is the measurement noise. The inverse problem is to estimate *S* given *Y* and *K*. Since the source dimension *N* is much larger than the number of electrodes *C*, making the ESI an ill-posed inverse problem, it is challenging to obtain a unique solution. Various regularization terms were designed based on the prior assumptions of the spatial and temporal structure of the source signal to find a unique solution. The inverse problem of ESI can be formulated as below:

1 $$\begin{aligned} S = {\mathop {\textrm{argmin}}\limits _S}\, \frac{1}{2} \left\| {Y - KS} \right\| _F^2 + \lambda R (S), \end{aligned}$$

where ${\Vert \cdot \Vert _F}$ is the Frobenius norm, and *S* can be obtained by solving the minimizing problem. The first term in Eq. (1) is the *data fitting* term trying to explain the recorded EEG/MEG measurements. The second term is called the *regularization* term, which is imposed to find a unique solution using sparsity or other neurophysiology-inspired regularizations. For example, if *R*(*S*) is a $\ell _2$ norm, the problem is called minimum norm estimate (MNE).

### Graph Fourier transform in brain source space

Consider an undirected graph $G = \left\{ \mathcal {V}, A\right\}$ generated from the 3D mesh of brain cortex, where $\mathcal {V} = \left\{ v_1, v_2, \ldots , v_N \right\}$ is the set of *N* nodes, *A* is the weighted adjacent matrix with entries given by the edge weights $a_{ij}$ that represents the connection strength between node *i* and node *j*. The graph Laplacian matrix is defined as $L = D - A$, where *D* is the degree matrix with $D_{ii} = \sum _{j\ne i}A_{ij}$. Since *L* is a positive semi-definite matrix, its eigenvalues are all greater or equal to 0, and the associated eigenvectors $U = [u_1, u_2, \ldots , u_N]$, $U \in \mathbb {R}^{N \times N}$ can be regarded as the basis vectors of GFT where any signal in the graph can be approximated as the linear combinations of basis. Thus, the GFT for a signal *S* can be defined as $\tilde{S}= U^{T}S$, whereas the inverse GFT is given as $S = U\tilde{S}$. Here we define normalized graph frequency (NGF) [27] as

2 $$\begin{aligned} f_G(u_i) = \frac{f_s(u_i)}{Tr(L)}, \end{aligned}$$

where *Tr*(*L*)is the trace of *L*, and $f_s(u_i)$ is defined as

3 $$\begin{aligned} f_s(u_i) = \sum _{m=1}^{N}\sum _{n\in \mathcal {N}(m)}\mathbb {I}(u_i(m)u_i(n)<0)/2, \end{aligned}$$

where $\mathcal {N}(m)$ represents all neighbors of node *m*, and $\mathbb {I}(\cdot )$ is the indicator function which equals 1 if the values of $u_i$ on node *m* and *n* have different signs and 0 otherwise. The number of sign flips at time *t* indicates how many zero crossings of a signal are within a bounded region at *t*.

**Fig. 1:**
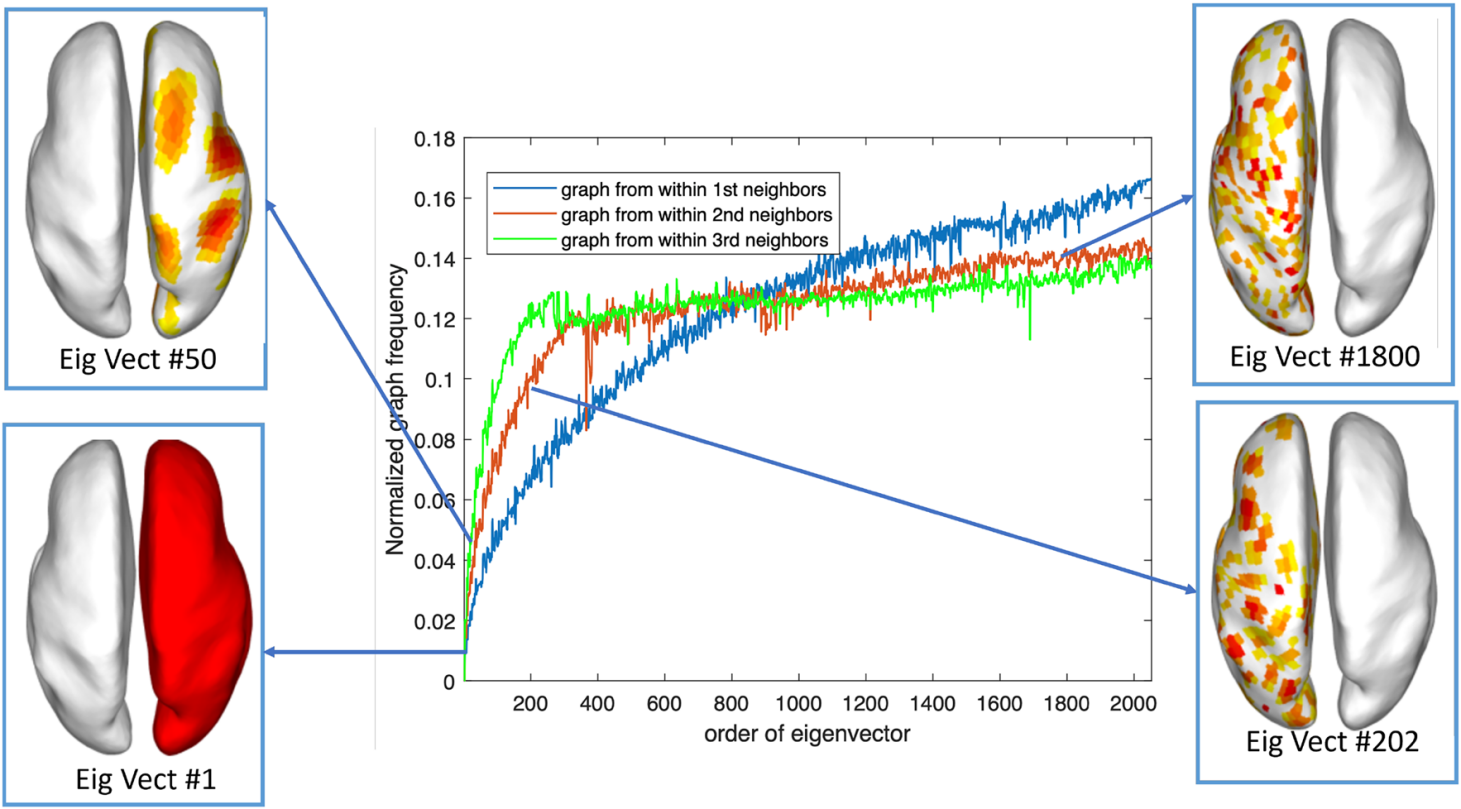
Source distributions corresponding to eigenvectors with different NGFs

We calculated the NGF in the whole time series within first-order neighbors, second-order neighbors, and third-order neighbors, respectively. The spectrogram, which is illustrated in Fig. 1, reveals that the NGF is positively correlated with the order of the eigenvalue of *L*. Thus we can further separate *U* into low, medium, and high-frequency components according to NGF values, and reformat it as $U = [U_{L}, U_{M}, U_{H}]$. The source activation patterns corresponding to eigenvectors with different NGFs are illustrated in Fig. 1.

### Brain source imaging with spatial graph filters

The existing sparse source localization methods with $\ell _1$-norm and $\ell _{2,1}$-norm as regularizations usually provide over-sparse solutions when estimating source extents, whereas the other non-sparsity methods commonly result in multiple foci. In this work, we propose to use the low-frequency subspaces spanned by the low-frequency graph filters (eigenvectors) to approximate an extended area of brain source localization. The proposed GFT technique can improve the performance of classical source localization methods by providing the low-frequency spatial graph filters $[U_{L}] \in \mathbb {R}^{N\times P}$, thus removing the high-frequency noises to reconstruct the focally extended sources. Here *P* serves as the cutoff value for the set of low-frequency components in the subspace. The intuition is that the main energy of the source signal usually lies in the low-frequency components which are associated with the regions on the cortex with relatively large source extend area in a time series. Keeping the low graph frequency could promote a source extend area reconstruction and decrease the impact of the noise. Moreover, the reduced dimensional estimation in the inverse problem could further constrain the solution space and make the solution more easily solved and robust.

Specifically, we project graph signals *S* onto a subspace spanned by $\tilde{U}$ with low NGF values. The data fitting term can be written as $\Vert Y- K\tilde{U}\tilde{U}^T S\Vert ^2_F$. Let $\tilde{S} = \tilde{U}^T S$ and $\tilde{K} = K \tilde{U}$, then the data fitting term can be rewritten as $\Vert Y- \tilde{K}\tilde{S}\Vert ^2_F$. Solving *S* is equivalent to finding parameters in the subspace $\tilde{U}$. Our goal is to estimate $\tilde{S}$, and all regularizations will be added to $\tilde{S}$ instead of *S*.

4 $$\begin{aligned} \hat{S}^*_{GFT} = {\mathop {\textrm{argmin}}\limits _{\tilde{S}^*}}\, \frac{1}{2} \Vert {Y - \tilde{K}\tilde{S}^*} \Vert _F^2 + \lambda R (\tilde{S}^*), \end{aligned}$$

Finally, the source estimation $\hat{S}_{GFT}$ can be simply obtained from the inverse GFT $\tilde{U}\hat{S}^*_{GFT}$.

We delineate the solutions for 5 classical ESI methods, including MNE [33], MCE [8], MxNE (with L21 implementation) [34], dSPM [6] and sLORETA [7], and show the solution or objective function of both original form and GFT-based form as follows.

*MNE* MNE is proposed by Hämäläinen by imposing an $\ell _2$ regularization on the source solution [33], the MNE algorithm has a closed-loop solution, given as

5 $$\begin{aligned} \hat{S}&= K^T(KK^T + \lambda I)^{-1} Y, \end{aligned}$$

We define $\tilde{K}=K\tilde{U}$, then the GFT-MNE solution is

6 $$\begin{aligned} \hat{S}_{GFT} = \tilde{U}\tilde{K}^T(\tilde{K}\tilde{K}^T + \lambda I)^{-1} Y. \end{aligned}$$

*sLORETA* sLORETA is a method proposed by Pascual-Marqui [35], and the solution is also in a closed form

7 $$\begin{aligned} \hat{S} = {(K^T(KK^T + \lambda I)^{-1}K)^{-\frac{1}{2}}} K^T(KK^T + \lambda I)^{-1} Y, \end{aligned}$$

The GFT-sLORETA solution is given as below:

8 $$\begin{aligned} \hat{S}_{GFT}=  \tilde{U} {(\tilde{K}^T(\tilde{K}\tilde{K}^T+ \lambda I)^{-1}\tilde{K})^{-\frac{1}{2}}} \\{} & {} \tilde{K}^T(\tilde{K}\tilde{K}^T + \lambda I)^{-1} Y. \end{aligned}$$

*dSPM* dSPM is proposed by Dale et al. It has a closed-form solution, and the solution is

9 $$\begin{aligned} \hat{S}=  {(K^T(KK^T + \lambda I)^{-1}I(K^T(KK^T + \lambda I)^{-1})^T)^{-\frac{1}{2}}} \\{} & {} K^T(KK^T + \lambda I)^{-1} Y, \end{aligned}$$

the GFT-dSPM solution can be obtained as:

10 $$\begin{aligned} \hat{S}_{GFT}= \,& {} \tilde{U} (\tilde{K}^T(\tilde{K}\tilde{K}^T + \lambda I)^{-1} \\{} & {} I(\tilde{K}^T(\tilde{K}\tilde{K}^T + \lambda I)^{-1})^T)^{-\frac{1}{2}} \\{} & {} \tilde{K}^T(\tilde{K}\tilde{K}^T + \lambda I)^{-1} Y. \end{aligned}$$

*MCE* MCE is a method proposed by Uutela et al. with $\ell _1$ regularization [8]. There is no closed-loop solution. Multiple iterative solvers can solve this problem. The objective function is:

11 $$\begin{aligned} \hat{S} = {\mathop {\textrm{argmin}}\limits _S}\, \frac{1}{2} \left\| {Y - KS} \right\| _F^2 + \lambda \Vert S\Vert _1, \end{aligned}$$

and the GFT-MCE solution can be obtained by solving the following equation:

12 $$\begin{aligned} \hat{S}_{GFT}^*= \,& {} {\mathop {\textrm{argmin}}\limits _S}\, \frac{1}{2} \left\| {Y - \tilde{K}S} \right\| _F^2 + \lambda \Vert S\Vert _1 \\ \hat{S}_{GFT}= & {} \tilde{U}\hat{S}_{GFT}^*. \end{aligned}$$

*MxNE* MxNE used a mixed norm as a regularization term, where $\ell _2$ norm is applied to the temporal domain and $\ell _p$ ($p=\{0.5,1\}$ is applied in the spatial domain, which is proposed by Gramfort et al. [34]. There is no closed-loop solution for this regularization. In this paper, we use $\ell _{2,1}$ norm version for MxNE. The objective function is:

13 $$\begin{aligned} \hat{S} = {\mathop {\textrm{argmin}}\limits _S}\, \frac{1}{2} \left\| {Y - KS} \right\| _F^2 + \lambda \Vert S\Vert _{2,1}, \end{aligned}$$

and the GFT-L21 solution is given as:

14 $$\begin{aligned} \hat{S}_{GFT}^*= & {} {\mathop {\textrm{argmin}}\limits _S}\, \frac{1}{2} \left\| {Y - \tilde{K}S} \right\| _F^2 + \lambda \Vert S\Vert _{2,1} \\ \hat{S}_{GFT}= & {} \tilde{U}\hat{S}_{GFT}^* \end{aligned}$$

where $\Vert \cdot \Vert _{2,1}$ denotes $\ell _2$ norm on the horizontal direction and $\ell _1$ on the vertical dimension of a matrix.

## Result

In this section, we conducted numerical experiments to validate the effectiveness of the proposed method on synthetic EEG data under different levels of neighbors (LNs) and signal Noise Ratio (SNR) settings for source localization followed by a causal time series reconstruction simulation. Lastly, we further validate it on real MEG recordings from a visual-auditory test.

### Simulation experiments

To validate the proposed GFT-based methods, We first conducted experiments on synthetic data with known activation patterns.

*Forward model* To generate synthetic EEG data, we build a realistic head model based on T1-MRI. The brain tissue segmentation and source surface reconstruction were conducted using FreeSurfer [36]. Then a three-layer boundary element method (BEM) head was built based on these surfaces, given the tissue conductivity values. A 128-channel BioSemi EEG cap layout was used, and the EEG channels were co-registered with the head model using Brainstorm [37] and then further validated on the MNE-Python toolbox [38]. The source space contains 1026 sources in each hemisphere, with 2052 sources combined, resulting in a leadfield matrix *L* with a dimension of 128 by 2052.

**Fig. 2:**
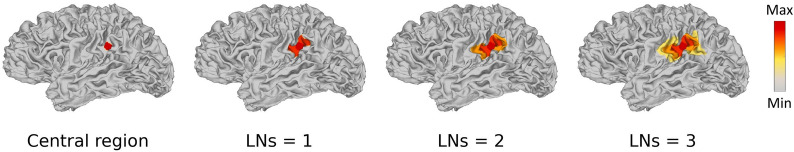
Brain source distributions with different levels of neighbors (LNs)

*Synthetic data generation* We randomly generated 200 source locations out of 2052 locations in the source space to conduct the experiments. Furthermore, as illustrated in Fig. 2, we used three different neighborhood levels (1-, 2-, and 3-level of the neighborhood) to represent different sizes of source extents, then we activated the whole “patch" with different neighborhood levels at the same time. The activation strength of the 1-, 2-, and 3-level adjacent regions was successively set to be 80%, 60%, and 40% of the central region. The scalp EEG data was generated based on the forward model under different measurement noise configurations with different Noise Ratio (SNR) set to be 40 dB, 30 dB, 20 dB, and 10 dB. SNR is defined as the ratio of the signal power $P_{\text {signal}}$ to the noise power $P_{\text {noise}}$: $\text {SNR}=10\log (P_{\text {signal}}/P_{\text {noise}})$. In total, there were 12: 3 (levels of neighborhood) $\times$ 4 (SNRs) data sets (*Y* and *S* pairs).

For causal time series reconstruction, we generated a simulated causal time series in the source space based on the Berlin Brain Connectivity Benchmark (BBCB) [39]. We slightly simplified it by using the autoregression with randomly generated 2 by 2 state transition matrix $\varvec{\Phi }$, with the order *K* to be 1 and the number of activated brain regions is set to be 2, to generate the source signal, given in Eq. 15.

15 $$\begin{aligned} x_t = \sum _{k=1}^K \varvec{\Phi }_k x_{t-k} + n_t, \ t=1,\ldots ,T,\ x_t\in \textbf{R}^N \end{aligned}$$

Then an independent random Gaussian noise with SNR from 10 to 40 dB was added at each time point. Lastly, a third-order Butterworth filter with zero phase delay was applied with pass bandwidth [0.1 Hz, 40 Hz].

*Experimental settings* We adopted MNE [5], MCE [8], $\ell _{2,1}$(MxNE) [34], dSPM [6], and sLORETA [7], as benchmark algorithms for comparison. We separately performed EEG source localization based on benchmark algorithms with and without the proposed GFT-based dimensionality reduction method. All the experiments were conducted on a Linux environment with CPU Intel(R) Xeon(R) Gold 6130 CPU @2.10 GHz and 128 GB memory.

*Source localization simulation results* The performance of each algorithm was quantitatively evaluated based on the following metrics:

1. *Localization error (LE)*: it measures the Euclidean distance between centers of two source locations on the cortex meshes.
2. *Area under curve (AUC)*: it is particularly useful to characterize the overlap of an extended source activation pattern.

**Fig. 3:**
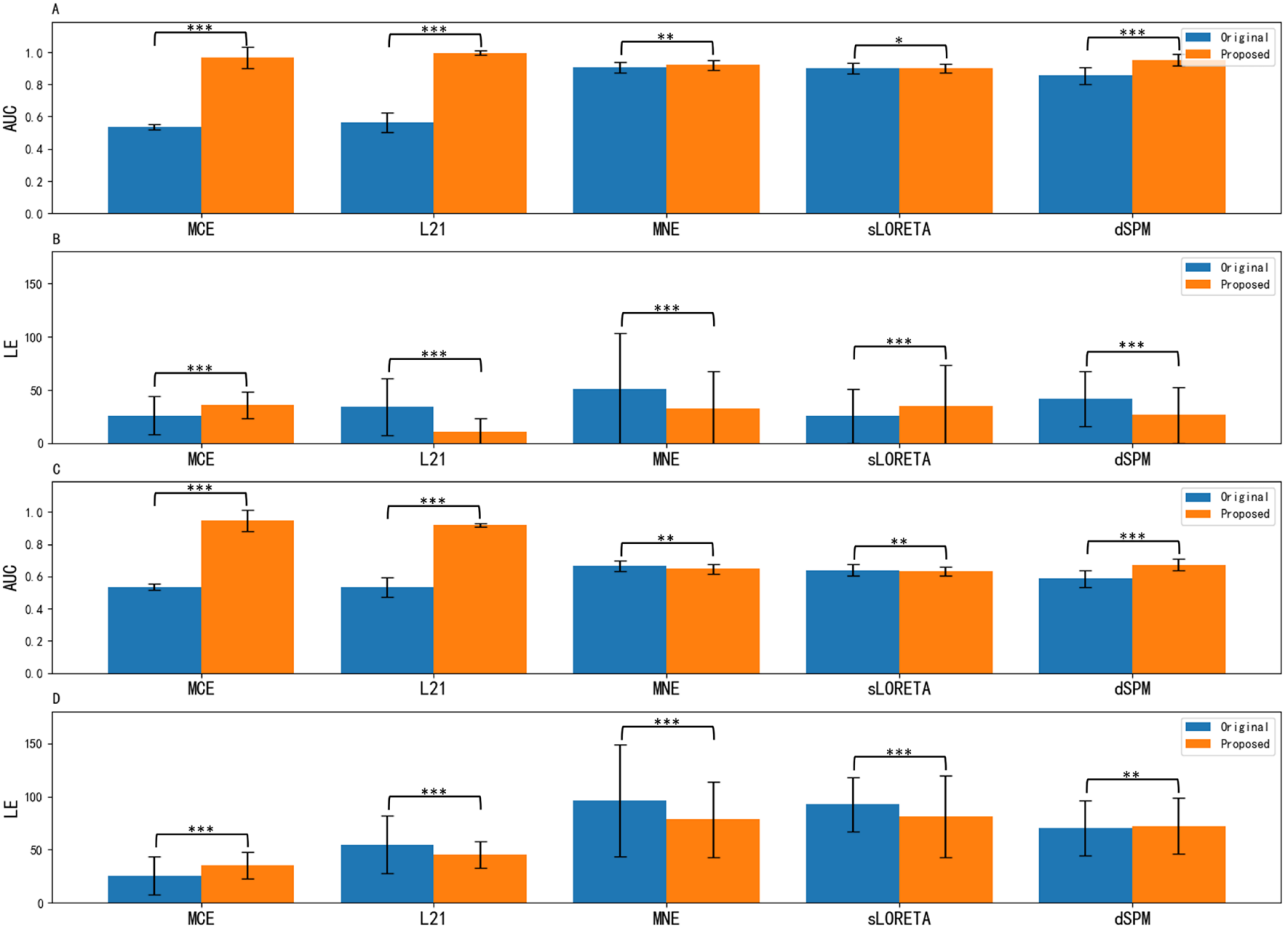
Significance of difference through t test on AUC and LE with 3-level of the neighborhood for SNR = 30 dB (subplot A and B), and SNR = 10 dB (subplot C and D). Asterisks indicate the results of paired-sample t tests: p value < 0.5(*), p value < 0.05(**), p value < 0.005(***)

**Fig. 4:**
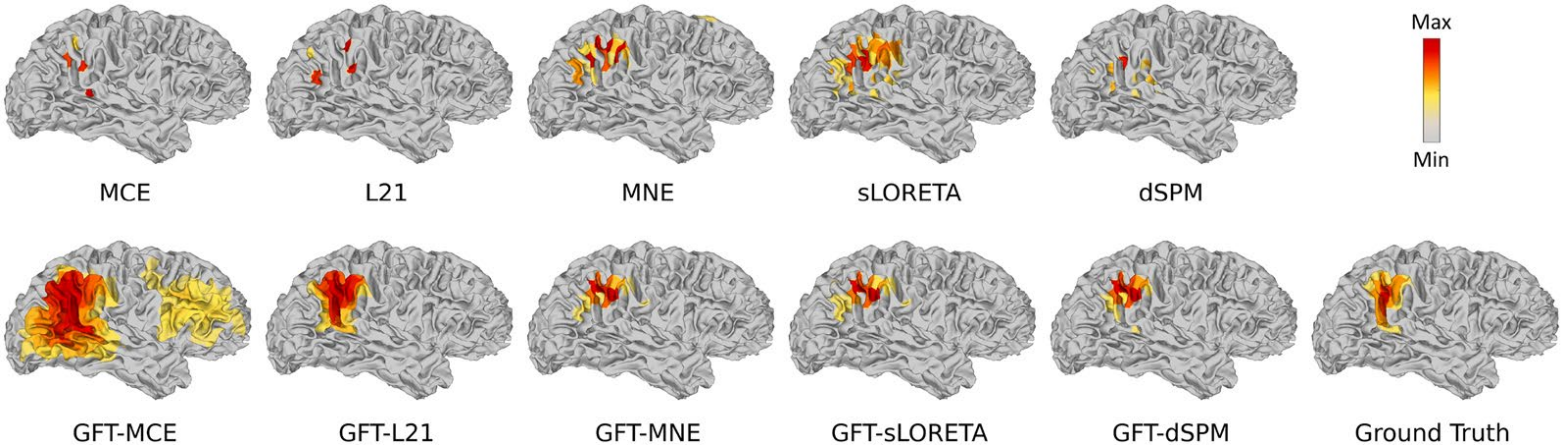
Brain sources reconstruction by different ESI algorithms with the single activated area and 3-level of the neighborhood for SNR = 40 dB

Better performance for localization is expected if LE is close to 0 and AUC is close to 1. The performance comparison between the proposed methods and benchmark algorithms on LE and AUC is summarized in Table 1, and the boxplot figures, as well as the difference significance test for SNR = 30 dB, and 10 dB are given in Fig. 3. The comparison between the reconstructed source distributions with a 3-level of the neighborhood and 40 dB SNR is shown in Fig. 4.

**Table 1:** Performance evaluation

SNR	Method	Source with LNs = 1		Source with LNs = 2		Source with LNs = 3	
		AUC	LE	AUC	LE	AUC	LE
40 dB	MCE	0.574 ± 0.059	19.667 ± 17.587	0.547 ± 0.030	23.715 ± 17.542	0.537 ± 0.018	25.770 ± 17.790
	GFT-MCE	0.865 ± 0.185	38.930 ± 14.862	0.940 ± 0.111	37.001 ± 13.258	0.957 ± 0.089	35.848 ± 12.438
	L21	0.557 ± 0.099	34.446 ± 28.195	0.560 ± 0.072	33.773 ± 26.604	0.559 ± 0.062	35.278 ± 27.603
	GFT-L21	0.977 ± 0.051	20.743 ± 16.816	0.994 ± 0.017	16.199 ± 17.748	0.996 ± 0.014	10.975 ± 13.137
	MNE	0.972 ± 0.034	29.069 ± 30.995	0.954 ± 0.032	32.367 ± 33.618	0.941 ± 0.028	34.224 ± 33.046
	GFT-MNE	0.991 ± 0.019	22.087 ± 26.172	0.967 ± 0.028	25.285 ± 25.978	0.951 ± 0.026	27.651 ± 26.083
	sLORETA	0.973 ± 0.034	9.952 ± 10.383	0.949 ± 0.031	15.208 ± 11.622	0.930 ± 0.027	18.155 ± 13.865
	GFT-sLORETA	0.989 ± 0.012	23.278 ± 26.710	0.958 ± 0.023	26.327 ± 26.425	0.940 ± 0.022	28.683 ± 26.543
	dSPM	0.952 ± 0.042	35.092 ± 25.358	0.916 ± 0.041	39.696 ± 27.028	0.888 ± 0.042	42.170 ± 26.496
	GFT-dSPM	0.995 ± 0.009	19.709 ± 23.126	0.985 ± 0.019	22.409 ± 22.882	0.970 ± 0.025	24.431 ± 22.872
30 dB	MCE	0.573 ± 0.059	19.729 ± 17.485	0.547 ± 0.030	23.910 ± 17.730	0.538 ± 0.018	26.087 ± 17.796
	GFT-MCE	0.873 ± 0.176	38.868 ± 14.806	0.951 ± 0.092	36.886 ± 13.232	0.968 ± 0.066	35.852 ± 12.462
	L21	0.567 ± 0.099	33.393 ± 27.697	0.567 ± 0.069	32.376 ± 26.137	0.563 ± 0.059	34.044 ± 26.876
	GFT-L21	0.978 ± 0.049	20.315 ± 16.254	0.995 ± 0.015	15.143 ± 15.930	0.997 ± 0.011	10.708 ± 12.695
	MNE	0.957 ± 0.045	38.450 ± 45.892	0.929 ± 0.040	42.829 ± 48.644	0.906 ± 0.034	50.860 ± 52.669
	GFT-MNE	0.990 ± 0.024	25.206 ± 33.390	0.951 ± 0.037	29.121 ± 34.989	0.920 ± 0.033	32.276 ± 35.514
	sLORETA	0.963 ± 0.046	16.588 ± 23.833	0.929 ± 0.041	22.422 ± 24.842	0.901 ± 0.035	25.689 ± 25.394
	GFT-sLORETA	0.984 ± 0.021	27.468 ± 35.861	0.934 ± 0.031	31.705 ± 37.753	0.902 ± 0.027	35.09 ± 38.352
	dSPM	0.940 ± 0.056	35.540 ± 25.774	0.891 ± 0.054	39.718 ± 26.799	0.854 ± 0.053	41.859 ± 25.901
	GFT-dSPM	0.995 ± 0.013	21.693 ± 26.864	0.977 ± 0.027	24.410 ± 27.060	0.953 ± 0.035	26.323 ± 26.260
20 dB	MCE	0.572 ± 0.058	19.426 ± 17.142	0.547 ± 0.030	23.403 ± 17.027	0.538 ± 0.018	25.204 ± 17.086
	GFT-MCE	0.879 ± 0.171	38.220 ± 14.542	0.953 ± 0.092	36.206 ± 12.959	0.969 ± 0.065	34.729 ± 11.946
	L21	0.567 ± 0.100	32.667 ± 29.531	0.569 ± 0.065	32.261 ± 28.750	0.565 ± 0.054	36.020 ± 31.831
	GFT-L21	0.980 ± 0.046	19.209 ± 15.773	0.996 ± 0.010	14.448 ± 16.017	0.997 ± 0.009	11.514 ± 17.229
	MNE	0.896 ± 0.069	70.686 ± 60.049	0.846 ± 0.057	83.812 ± 58.453	0.807 ± 0.046	92.826 ± 55.580
	GFT-MNE	0.957 ± 0.053	36.198 ± 43.792	0.868 ± 0.056	45.584 ± 47.039	0.806 ± 0.046	56.759 ± 49.333
	sLORETA	0.914 ± 0.073	47.694 ± 51.549	0.851 ± 0.062	61.623 ± 54.220	0.802 ± 0.052	76.219 ± 54.994
	GFT-sLORETA	0.931 ± 0.063	41.632 ± 48.490	0.833 ± 0.053	53.106 ± 51.408	0.776 ± 0.043	65.194 ± 52.521
	dSPM	0.880 ± 0.094	37.385 ± 27.315	0.799 ± 0.089	40.268 ± 27.599	0.740 ± 0.081	43.687 ± 28.075
	GFT-dSPM	0.977 ± 0.039	28.313 ± 33.943	0.922 ± 0.054	32.615 ± 34.334	0.865 ± 0.058	39.456 ± 35.939
10 dB	MCE	0.568 ± 0.057	20.197 ± 17.414	0.545 ± 0.029	24.231 ± 17.784	0.535 ± 0.017	25.581 ± 17.447
	GFT-MCE	0.863 ± 0.188	38.663 ± 14.157	0.930 ± 0.128	36.489 ± 12.496	0.947 ± 0.110	35.271 ± 11.621
	L21	0.546 ± 0.088	40.739 ± 39.937	0.538 ± 0.057	47.475 ± 44.186	0.534 ± 0.043	54.949 ± 47.103
	GFT-L21	0.971 ± 0.076	29.000 ± 33.911	0.982 ± 0.046	35.873 ± 46.373	0.922 ± 0.077	45.569 ± 50.570
	MNE	0.731 ± 0.138	92.912 ± 48.956	0.693 ± 0.104	94.861 ± 46.006	0.665 ± 0.089	96.173 ± 45.351
	GFT-MNE	0.765 ± 0.137	69.890 ± 42.483	0.684 ± 0.096	76.526 ± 38.688	0.647 ± 0.082	78.559 ± 36.579
	sLORETA	0.726 ± 0.135	89.228 ± 48.313	0.673 ± 0.102	91.018 ± 44.866	0.640 ± 0.087	92.693 ± 44.886
	GFT-sLORETA	0.737 ± 0.130	74.17 ± 44.229	0.662 ± 0.094	79.575 ± 39.930	0.633 ± 0.082	80.937 ± 38.260
	dSPM	0.682 ± 0.155	60.615 ± 43.931	0.619 ± 0.12	66.894 ± 44.070	0.586 ± 0.101	70.274 ± 43.919
	GFT-dSPM	0.797 ± 0.142	61.567 ± 36.941	0.719 ± 0.101	68.552 ± 34.038	0.673 ± 0.082	72.215 ± 32.479

From Table 1 and Figs. 3 and 4, we can find that: (1) MNE, MCE, $\ell _{2,1}$, sLORETA, and dSPM can only reconstruct the brain sources when the activated area is small and the SNR level is high, and even the evaluation metrics can be good in some cases, the reconstruction for source extend area is discrete or disconnected as shown in Fig. 4. As the source range expands and the SNR decreases, a significant increase in LE and an obvious reduction in AUC can be observed. The reconstructed source distributions are no longer concentrated. (2) By contrast, the results of the proposed GFT-based methods outperform benchmark methods in most cases after applying the spatial graph filters. They both show good stability for varied neighborhood levels and SNR settings. Particularly, the performance of the proposed GFT-based $\ell _1$ regularization family (i.e., $\ell _1$-norm and $\ell _{2,1}$-norm) exhibits better performance on reconstructing source extents without losing its advantage in sparse focal source reconstruction and outperforms other methods in most instances.

**Fig. 5:**
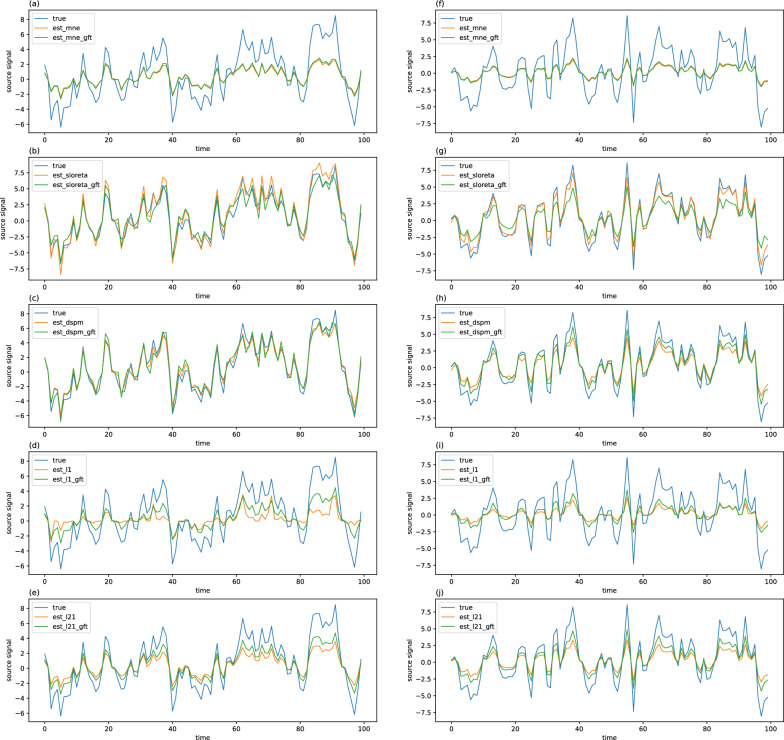
Time series reconstruction on activated region at SNR = 20 dB, where the sub-figures (a)–(e) on the left column are the reconstructions of the original and proposed GFT-based forms of MCE, L21, MNE, sLORETA, and dSPM at casual source 1, whereas the sub-figures (f)–(j) on the right column are the same definition at casual source 2. The blue curves are true source signals, and the orange as well as green curves are original and proposed GFT-based forms of different methods respectively

*Causal time series reconstruction* An order *K* = 1 causal time series was generated at different levels of noise. As the reconstructed time series at the source space is impacted by the strength of regularizations, to rectify this impact, we use the correlation of two sets of time series to quantify the similarity between the reconstructed time series and the ground true time series. A detailed comparison of different methods is given in Table 2, and an example of source reconstruction at 20 dB SNR is illustrated in Fig. 5.

**Table 2:** Pearson correlation for causal time series reconstruction

Methods	SNR = 10 dB	SNR = 20 dB	SNR = 30 dB	SNR = 40 dB
MCE	0.575 ± 0.276	0.591 ± 0.287	0.595 ± 0.287	0.597 ± 0.286
GFT-MCE	0.904 ± 0.115	0.908 ± 0.127	0.907 ± 0.143	0.907 ± 0.144
L21	0.847 ± 0.335	0.888 ± 0.281	0.888 ± 0.285	0.887 ± 0.286
GFT-L21	0.976 ± 0.051	0.980 ± 0.048	0.980 ± 0.047	0.980 ± 0.047
MNE	0.909 ± 0.095	0.973 ± 0.066	0.981 ± 0.066	0.982 ± 0.066
GFT-MNE	0.910 ± 0.093	0.974 ± 0.061	0.982 ± 0.061	0.983 ± 0.062
sLORETA	0.912 ± 0.100	0.970 ± 0.076	0.977 ± 0.076	0.978 ± 0.076
GFT-sLORETA	0.890 ± 0.112	0.968 ± 0.080	0.979 ± 0.080	0.980 ± 0.080
dSPM	0.911 ± 0.115	0.965 ± 0.093	0.971 ± 0.093	0.972 ± 0.093
GFT-dSPM	0.921 ± 0.088	0.973 ± 0.069	0.979 ± 0.070	0.980 ± 0.070

It is worth noting that the proposed GFT-based methods are better than their original counterparts in most cases. Even at low SNR levels, the proposed methods can also perform well. Particularly, the GFT-based $\ell _1$ regularization family has a significant performance improvement compared to that of their original form which is consistent with the findings in the source localization simulation above, and this phenomenon can be observed in Fig. 5, where the reconstruction results of the GFT-based $\ell _1$ regularization family method are significantly better than the original method, especially the reconstruction of the source signal trend during some time intervals.

### Real data experiments

**Fig. 6:**
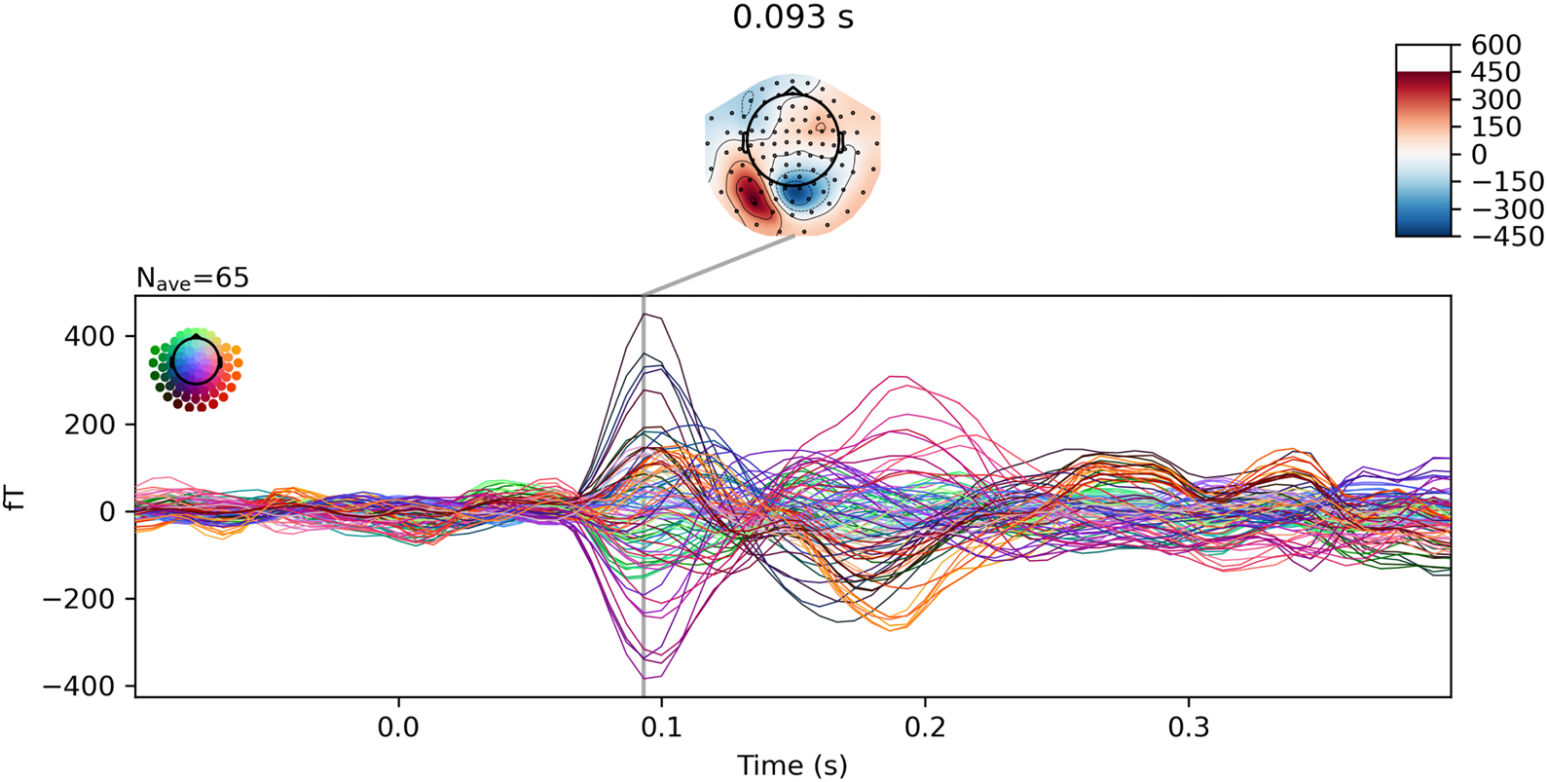
Averaged MEG time series plot and topographies

**Fig. 7:**
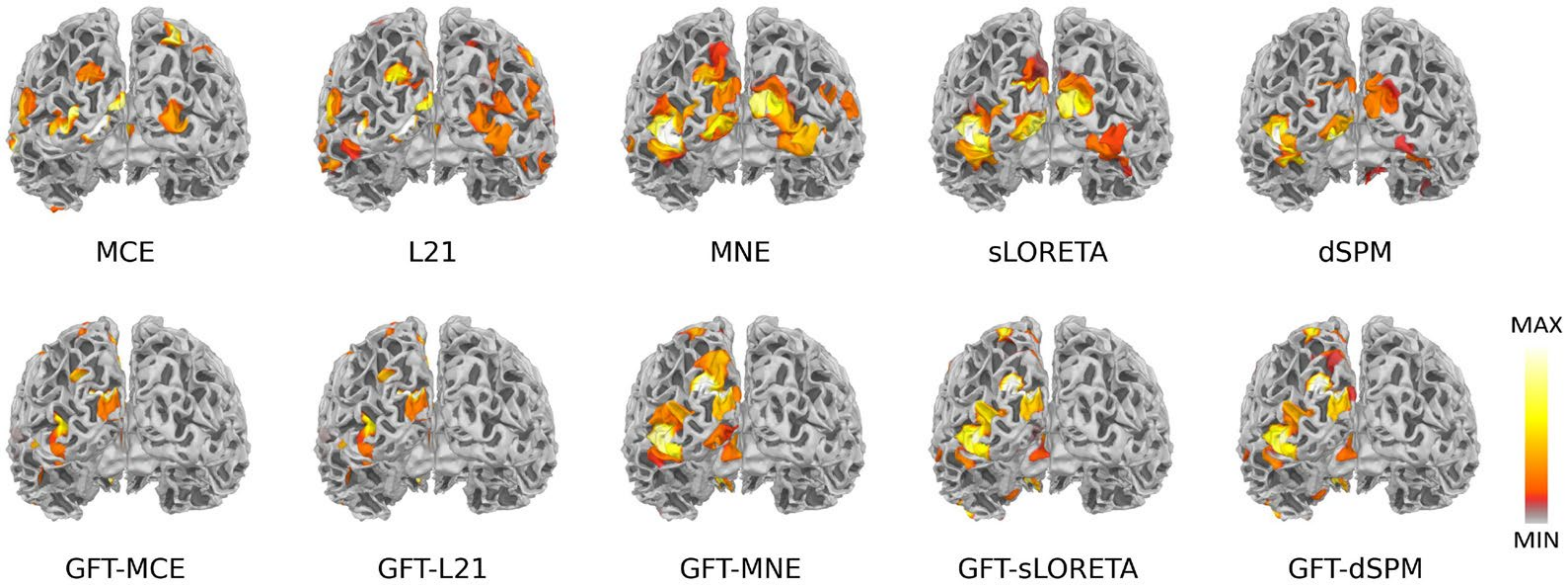
Reconstructed source activation patterns from MEG data

We further validated the proposed methodology on a real dataset that is publicly accessible through the MNE-Python package [38]. In this dataset acquirement, checkerboard patterns were presented into the left and right visual field, interspersed by tones to the left or right ear with a stimuli interval of 750 ms. The subject was asked to press a key with the right index finger as soon as possible after the appearance of a smiley face was presented at the center of the visual field [40]. Evoked Response Potentials (ERP) were extracted from the MEG measurements, and then we averaged these ERPs for source reconstruction under MNE, MCE, $\ell _{2,1}$, dSPM, sLORETA with and without the proposed GFT operations. The averaged spikes are shown in Fig. 6, and the reconstructed source distributions are shown in Fig. 7.

From Fig. 7, we can see that the source area estimated by MNE, MCE, $\ell _{2,1}$, sLORETA, and dSPM is highly broad. By contrast, the proposed GFT-based methods provide more sparse focal source reconstructions. Moreover, the reconstructed focal for the proposed GFT-based methods falls primarily on areas with the strongest source signal, while others would spread to several regions. Obviously, the spatial graph filter in the proposed GFT-based methods promotes a concentrated and accurate estimation of the visual zone.

## Discussion

We showed that the subspace composed of low-frequency spatial graph filters can be used to approximate the compact and extended source activation patterns, which is a non-parametric approach, thus empowering the classical ESI methods to have better reconstruction performance for source extents reconstruction. This approach is an easy and effective pre-processing technique compared to predefined spatial gradient based methods or data-driven methods in a deep learning paradigm. While we validated 5 classical methods, it is worth noting that most of the other classical approaches, such as recursive multiple signal classifier (MUSIC), recursively applied and projected MUSIC (RAP MUSIC), low-resolution brain electromagnetic tomography (LORETA), FOCUSS, weighted minimum norm (WMN), shrinking LORETA-FOCUSS, hybrid weighted minimum norm method, recursive sLORETA-FOCUSS, standardized shrinking LORETA-FOCUSS (SSLOFO), etc., can use the proposed GFT in the source space before applying each specific method.

## Conclusion

In this study, we improved the classical source localization methods by using spatial graph filters to solve the inverse problem of ESI. The proposed methodology enjoys the advantage of reconstructing focal source extents with sparsity and minimizing the impact of the noise by transforming the estimation of the source signal into a projected subspace spanned by spatial frequency graph filters. Numerical experiments demonstrated that the proposed method performs particularly well on source extents, yields excellent robustness when the SNR level is low, and can better reconstruct the source time series, specifically the performance of the $\ell _1$ family regularization can be greatly improved with the GFT. In the experiment on real data we performed, the proposed methodology provides a satisfactory reconstruction with more concentrated source distribution and more stability to noise than benchmark algorithms.

## Data Availability

The public data used can be accessed through: https://mne.tools/stable/index.html. The code will be accessible upon request to the corresponding authors.
